# Predictors of durable no evidence of disease status in de novo metastatic inflammatory breast cancer patients treated with neoadjuvant chemotherapy and post-mastectomy radiation

**DOI:** 10.1186/2193-1801-3-166

**Published:** 2014-03-31

**Authors:** Vinita Takiar, Catherine L Akay, Michael C Stauder, Welela Tereffe, Ricardo H Alvarez, Karen E Hoffman, George H Perkins, Eric A Strom, Thomas A Buchholz, Naoto T Ueno, Gildy Babiera, Wendy A Woodward

**Affiliations:** Department of Radiation Oncology, The University of Texas MD Anderson Cancer Center, Houston, TX USA; Department of Surgical Oncology, The University of Texas MD Anderson Cancer Center, Houston, TX USA; Department of Breast Medical Oncology, The University of Texas MD Anderson Cancer Center, Houston, TX USA; Morgan Welch Inflammatory Breast Cancer Research Program and Clinic, The University of Texas MD Anderson Cancer Center, Houston, TX USA; Department of Radiation Oncology–Unit 97, The University of Texas MD Anderson Cancer Center, 1515 Holcombe Boulevard, Houston, TX 77030 USA

**Keywords:** Post-mastectomy, Radiation therapy, Inflammatory breast cancer, Metastatic disease, Pathologic complete response

## Abstract

**Introduction:**

Definitive locoregional therapy including surgery and post-mastectomy radiation therapy (PMRT) has been offered to select IBC patients with de novo metastatic disease. Herein we examined predictive factors for progression-free survival after comprehensive PMRT radiation +/- locoregional treatment of metastatic sites.

**Methods:**

Charts of T4d, any N, M1 (de novo) patients who completed PMRT to ≥ 50 Gy from 2006–2011 were reviewed. Patients who received doses <50Gy to the primary site, received radiation at another facility or were treated pre-operatively were excluded. The remaining 36 patients formed the study cohort. Progression-free survival post-PMRT (PFSx) was assessed from the last day of radiation. Median dose to primary fields was 51 Gy. Boost doses ranged from 6–16 Gy.

**Results:**

Median age at diagnosis was 54 (range 33–70). Median follow up from primary irradiation completion was 31 months. Sixteen patients were Stage IV NED at last follow-up (IR 37–60 mo). Fifteen patients died of disease. Five patients experienced an in-field recurrence, three of which resulted from local recurrence at the medial edge of the field. Actuarial 5 year locoregional control (LRC) was 86%. Median PFSx was 20 months. All sites of gross disease were treated with radiation in 21/36 patients. Location of metastatic disease had no correlation with PFSx. Estrogen receptor (ER)- patients had shorter 5-yr actuarial PFSx (28% vs. 66%, P = 0.03) and 5 year actuarial OSx (37% vs 71%, P = 0.02). Nine patients (25%) developed a pathological complete response (pCR) after chemotherapy and with a median follow-up of 59 months, 7 remained without evidence of disease.

**Conclusions:**

Despite the poor prognosis associated with metastatic IBC, our data suggest that select patients may be appropriate candidates for locoregional therapy. Patients who achieve a pCR or those with ER + disease have a favorable PFSx. It remains unclear whether all gross disease needs to be addressed with locoregional therapy to provide benefit.

## Introduction

Historically, a diagnosis of inflammatory breast cancer (IBC) was uniformly fatal with 5 year overall survival (OS) rates <5% in multiple studies in the 1970s with a median survival of 1.2 years. (Robbins et al. 1974; Stocks and Patterson 1976; Zucali et al. 1976) However, the advent of polychemotherapy and endocrine therapy has led to improved survival as demonstrated in large meta-analyses as well as in our large (n = 398) single institutional experience of patients treated for IBC, with 5 year OS reported to be 46.1% at a median follow up of 5.8 years. (Early Breast Cancer Trialists’ Collaborative Group 1988,1998; Gonzalez-Angulo et al. 2007) These values stand in stark contrast to the 5 year OS of 79.2% reported for a contemporary population of non-metastatic breast cancer patients treated with multimodality therapy (Greenbaum et al. 2010). Although IBC is still considered to be the most aggressive form of breast cancer, there have clearly been improvements in treatment paradigms over the decades (Anderson et al. 2003). However, given the relative rarity of IBC (1–3% of breast cancers), there is limited data on stage IV (de novo) IBC at presentation, and essentially none on the role of aggressive radiation therapy in this setting (Wingo et al. 2004).

IBC is a clinical diagnosis, encompassing the rapid onset of diffuse erythema and edema of the breast in the absence or presence of a discrete mass (AJCC 2010). By definition, the symptoms must have developed over <6 months and involve >1/3 of the breast. Additional presenting symptoms can include pain, tenderness, and ulceration (Jaiyesimi et al. 1992). Although dermal lymphatic invasion is a characteristic pathologic finding, it is not required for diagnosis (Dawood et al. 2011).

Patients with non-metastatic IBC face the prospect of a lengthy definitive treatment course, consisting of neoadjuvant chemotherapy, modified radical mastectomy, post-operative radiation therapy, and adjuvant systemic therapy, if warranted. Neoadjuvant chemotherapy forms the cornerstone of treatment, demonstrating improved disease-free and overall survival for IBC patients (Ueno et al. 1997). Pathologic response in the breast and lymph nodes is considered to be highly prognostic (Buzdar et al. 1995; Rouesse et al. 1986; Rouzier et al. 2002). In a series of 54 patients with IBC, 10 yr OS was 35% in all patients, however if there was pCR after neoadjuvant chemotherapy, 50% of patients achieved 10 year DFSx. Lymph node involvement is also considered to be prognostic(Jaiyesimi et al. 1992; Lerebours et al. 2003) as is extensive erythema, negative hormone receptor status, and p53 gene mutation (Chevallier et al. 1987; Riou et al. 1993). Given that lymph node metastases are therefore both common and prognostic, the benefit of neoadjuvant chemotherapy is attributed to the ability to address subclinical sites of metastatic disease with the underlying hypothesis that IBC likely represents a systemic process from the start. This logic is further supported by studies such as that by Tabanne et al. who note metastases within 2 months of locoregional treatment, despite no evidence of metastatic (M1) disease on initial staging workup (Tabbane et al. 1977).

Pending a response to neo-adjuvant systemic treatment, patients are then considered for modified radical mastectomy prior to consolidative radiation therapy, which has a demonstrated locoregional control (LRC) benefit (Fleming et al. 1997; Schafer et al. 1987). However, this multimodality treatment paradigm is typically not applied to all patients with M1 disease that could be feasibly and safely eradicated by local therapy. Traditionally, M1 disease, Stage IV, has been treated by systemic therapy alone with palliative local therapy as indicated. However, in the setting of effective systemic treatment to address distant subclinical sites of disease and feasible options for consolidative local therapy (radiation and surgery), the definitive paradigm has been applied in select cases presenting with Stage IV IBC. A recent retrospective report by Akay et al. including 172 cases of metastatic IBC reports increased overall survival and distant progression-free survival in those patients who received chemotherapy along with radiotherapy and surgery in comparison to those that received chemotherapy with either surgery or radiotherapy alone (Akay et al. 2014).

Here, we review in greater detail the subset of these patients representing a contemporary, single-institutional cohort of women treated for Stage IV IBC, in a dedicated multi-disciplinary IBC clinic, with neoadjuvant chemotherapy, followed by definitive locoregional treatment, comprised of surgery and post-mastectomy radiation therapy to ≥50 Gy. Study objectives included evaluation of clinical outcomes in this population subset as well as identification of factors to guide medical decision making in these challenging clinical scenarios.

## Methods

### Patients

This study was approved by the Institutional Review Board at The University of Texas, MD Anderson Cancer Center. The institution and State of Texas dedicated resources to an IBC clinic in 2006. We retrospectively reviewed medical records for all patients diagnosed with T4d Nany M1 (de novo) inflammatory breast cancer from 2006–2011. Only those patients who were found to have metastases within 3 months of IBC diagnosis were considered. Sixty-four percent of these patients (n = 117) did not receive radiation therapy or surgery. Fifty-three patients were identified who had completed post-mastectomy radiation therapy (PMRT). Sixteen patients who received palliative radiotherapy to <50 Gy to the primary site or were treated pre-operatively were excluded. An additional patient who received prior radiotherapy at an outside facility and was reirradiated at our institution was also excluded. The remaining 36 patients form the current study population.

Prior to treatment, all patients underwent appropriate staging workup as well as multidisciplinary consultation in our Breast Center. Medical photographs were taken prior to chemotherapy to guide eventual radiation field design. All patients had pathology confirmed by our in-house pathologist. Metastatic sites were confirmed by ultrasound or CT-guided biopsies when appropriate and feasible. All patients in this series received neoadjuvant chemotherapy, consisting of weekly Taxol for 12 weeks followed by either FAC (5-fluorouracil, Adriamycin, and cyclophosphamide) or FEC (5-fluorouracil, epirubicin, and cyclophosphamide) every three weeks for four cycles, per institutional practice. All estrogen receptor (ER) or HER-2/*neu* positive patients received appropriately targeted therapies under the supervision of a medical oncologist.

After modified radical mastectomy, no evidence of disease by pathology review of the breast and lymph nodes was considered to be a pathologic complete response (pCR). Pathologic CR does not imply that a site of metastatic disease was resected. Review of post-chemotherapy imaging at the time of consultation for comprehensive post-mastectomy radiation therapy was considered a radiographic complete response (rCR).

Pre-chemotherapy cross-sectional imaging was used to delineate target volumes. A combination of 6 and 18 MV photons were used to treat the chest wall and axillae as required to provide sufficient dose to the target volume. Clinically appropriate electron energies were chosen to treat the supraclavicular and internal mammary lymph nodes chains. An appositional photon field with a half-beam block was used to treat the supraclavicular fossae. Electron supplements were also used to boost nodal basins that were involved prior to chemotherapy and not oncologically dissected, to a definitive dose.

Of the 36 patients, 34 were treated exclusively with a 3D conformal approach using a combination of photons and electrons. One patient received radiation therapy to the supraclavicular fossa with intensity-modulated radiation therapy (IMRT) to allow for concurrent radiation therapy to her metastatic disease in the C5 vertebral body. A second patient received proton beam radiation therapy to reach her anterior mediastinal metastasis but minimize lung dose. All patient histories and treatment plans were reviewed at the Breast Radiation Oncology Quality Assurance meeting. Tissue equivalent bolus schedules varied depending on fractionation and dermatitis.

Follow up for each patient consisted of visitation with the medical oncologist to discuss further systemic treatment options if appropriate, as well as appointments with the treating radiation oncologist at least once every 2–4 months for the first two years after completing treatment, with appropriate imaging, or earlier if there were concerns about radiation-induced complications or disease progression. Patients were seen every 4–6 months thereafter. NED status was based on radiographic reports. All locoregional recurrences (LRR), in the chest wall or ipsilateral draining lymphatic nodal basins, as identified by diagnostic imaging or physical examination were confirmed by biopsy.

### Data analysis

Progression-free survival was assessed from the last day of radiation therapy to the primary site. NED status was based on response to radiation therapy, metastasectomy or systemic treatment. Patients with no evidence of local or distant disease, based on clinical and radiographic information, at last follow up, were deemed to be progression-free, stage IV NED. All actuarial Kaplan-Meier and log rank statistical analyses were performed using SPSS Statistics 21.0 (IBM Corporation).

## Results

### Patient characteristics

All 36 patients had clinical presentations consistent with T4d, inflammatory breast cancer using the international consensus definition (AJCC 2010). Detailed patient characteristics are presented in Table 1. Median age at time of diagnosis was 54 years (range 33–70 years). Twenty-four patients had M1 disease involving lymph nodes. Of the 16 patients that were ER+, 15 were prescribed endocrine therapy. Of the 36 patients, 19 (53%) underwent biopsy of the metastatic site. Two of these patients (with M1 disease in the contralateral supraclavicular area and retroperitoneal area) were found to have negative biopsies which were considered to be falsely negative in the setting of substantial radiologic evidence of disease. Of the patients that did not undergo biopsy to their site of M1 disease, the largest fraction (8 patients) had bone involvement.

**Table 1:** **Patient Characteristics**

Characteristic	N (% of total 36 patients)
*Age at diagnosis*	
30–39	3 (8)
40–49	9 (25)
50–59	13 (36)
60–69	10 (28)
70–79	1 (3)
*Race*	
White	29 (81)
Black	4 (11)
Hispanic	2 (6)
Asian	1 (3)
*Receptor Status*	
ER+, H2N-	12 (33)
ER-, H2N-	10 (28)
ER-, H2N+	8 (22)
ER+, H2N+	6 (17)
*Site of M1 disease*	
Bone	9
Liver	5
Lung	3
Contralateral axillary LN	11
Mediastinal LN	8
Contralateral SCV LN	3
Contralateral Cervical LN	1
Retroperitoneal LN	1
Arm	1
Ovary	1
*Radiation Sites*	
PMRT +/- select M1	13(36)
PMRT + All M1	23(64)
*Regional LN involved*	
Internal mammary LN	6
Infraclavicular LN	17
Supraclavicular LN	13

### Treatment properties

Neoadjuvant chemotherapy was followed by modified radical mastectomy and radiation therapy to the chest wall and ipsilateral regional lymph node basins and M1 sites when feasible and safe. Patients generally received treatment to 50–54 Gy in 2 Gy per fraction (22 patients) or 51 Gy in 1.5 Gy fractions twice daily (14 patients). The patients treated twice daily met at least one high risk criteria including poor response to neoadjuvant systemic treatment, positive margins after surgery, or age <45 years (Bristol et al. 2008). The mastectomy scar and chest wall were then boosted with appropriate electron energy an additional 10 Gy to 15 Gy dependent on fractionation. Fifteen patients received a boost to the infraclavicular fossa ranging from 9 Gy to 16 Gy. Nineteen patients underwent a boost to the supraclavicular lymph nodes with doses ranging from 6 to 16 Gy as deemed clinically appropriate by the treating physician. Two patients received concurrent chemotherapy with capecitabine (825 mg/m^2^ twice daily).

Of the 36 patients, 18 had a radiographic complete response (rCR) to neoadjuvant systemic treatment. Of these patients, nine patients (50%) had a pathologic complete response on evaluation of the breast and lymph node tissue. For 23 of the 36 patients, all sites of metastatic disease were addressed by local therapy (surgery and/or radiation therapy), with 21 of these patients receiving radiation to all sites of metastatic disease. Two patients, with ovarian metastatic disease and liver metastasis underwent surgery to address their sites of M1 disease.

### Disease control

**Figure 1:**
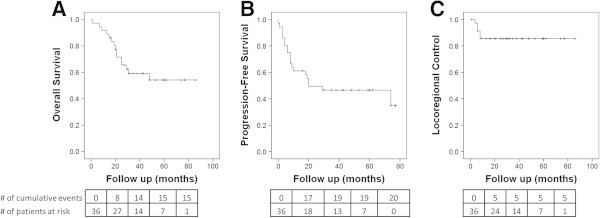
**Actuarial disease outcomes for patients treated with neoadjuvant chemotherapy, modified radical mastectomy, and post-mastectomy radiation therapy. (A)** overall survival rate, **(B)** progression-free survival after PMRT, and **(C)** locoregional control rate.

Median follow up from primary radiation therapy completion was 31 months (interquartile range [IR] 18–55 months). At 2 years follow up, actuarial OSx was 71%, PFSx after PMRT was 50%, and LRC was 86% (Figure 1). At 5 years, these values were 54%, 47%, and 86% respectively. In total, 20 patients experienced disease progression, with 15 patients dying of their disease. There were 5 local recurrences. Three of these patients failed at the medial border of the treatment field, with one of these patients recurring broadly, and a second patient also presenting with disease recurrence inferior to the field. Among the final two patients who experienced a local chest wall recurrence, one was centered within the field and one occurred prior to radiation therapy commencement. Of all patients, sixteen (44%) were Stage IV with no evidence of disease (NED) as of last follow-up (median follow-up in this cohort was 51 months with IR 37–60 months). Characteristics of this subset of patients are presented in Table 2.

**Table 2:** **Characteristics of sixteen patients currently NED**

Characteristic	N (% of 16 patients)
*Receptor Status*	
ER+, H2N-	5 (31)
ER-, H2N-	2 (13)
ER-, H2N+	3 (19)
ER+, H2N+	6 (38)
*Radiation Sites*	
PMRT +/- select M1	6 (38)
PMRT + All M1	10 (62)
*Site of M1 disease*	
Bone	4 (25)
Lung	2 (13)
Contralateral axillary LN	3 (19)
Mediastinal LN	3 (19)
Contralateral SCV LN	1 (6)
Retroperitoneal LN	1 (6)
Ovary	1 (6)
Liver	1 (6)
*Response*	
pCR	7 (44)
rCR or pCR	9 (56)
no pCR	9 (56)

### Prognostic factors for durable NED

**Figure 2:**
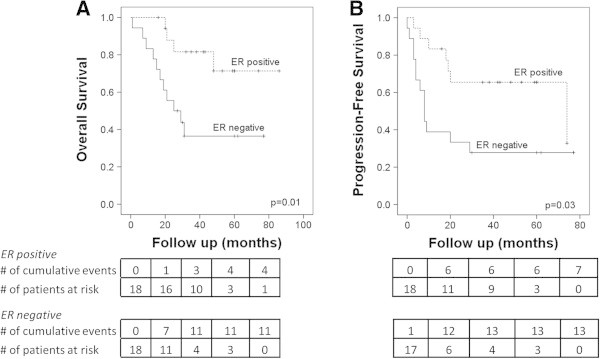
**Estrogen receptor positivity results in improved outcomes in Stage IV IBC patients after PMRT. (A)** overall survival **(B)** progression-free survival.

**Figure 3:**
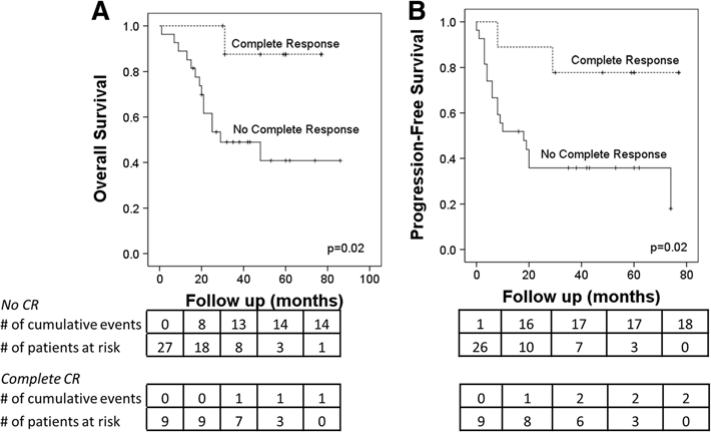
**Pathologic complete response to neoadjuvant chemotherapy results in improved outcomes in Stage IV IBC patients after PMRT. (A)** overall survival **(B)** progression-free survival.

**Figure 4:**
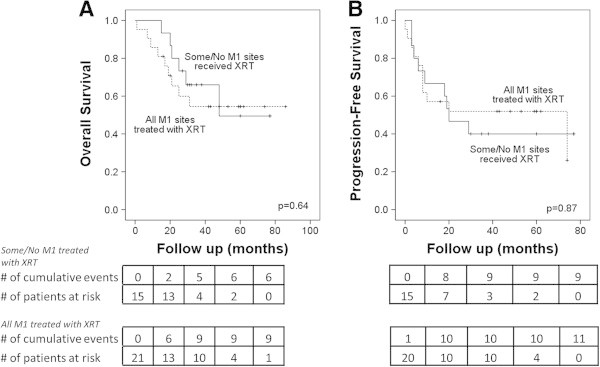
**Treating all sites of metastatic disease with radiation therapy does not result in improved outcomes in Stage IV IBC patients after PMRT. (A)** overall survival and **(B)** progression-free survival.

To evaluate for prognostic factors that may predict for improved outcome, patients were stratified into subgroups for further analysis. Five-year actuarial OSx and PFSx were significantly lower among those patients whose disease was ER negative (OSx: 37 months vs 71 months, p = 0.02; PFSx: 28 vs 66 months, p = 0.03) (Figure 2). Her-2/neu positivity did not significantly affect 5 year OSx (p = 0.07) or PFSx (p = 0.10). In patients who had a pathologic complete response (pCR) to systemic treatment, 5 year OSx was significantly improved as shown in Figure 3A (88% vs 40%; p = 0.02) as was 5 year PFSx as shown in Figure 3B (78% vs 36%, p = 0.02). Of 9 patients who achieved pCR, 7 remained without evidence of disease at last follow up. OSx and PFSx were no longer significantly different if patients with complete radiographic response to systemic treatment were included in the good response cohort (p = 0.08, p = 0.30 respectively). Comparing patients who could safely and feasibly receive radiation therapy to all sites of metastatic involvement, with those who did not receive radiation to all sites, there was no difference in OSx or PFSx (p = 0.64, p = 0.87 respectively) as demonstrated in Figure 4. Location of metastatic disease had no effect on OSx (p = 0.67) or PFSx (p = 0.49) by log-rank analysis.

For hypothesis generation, we further examined the outcome by response among PMRT patients for who all metastatic deposits could be safely irradiated versus those in whom only what could be encompassed feasibly was irradiated. Although the numbers are small, examining the nine patients who had a pCR, eighteen who had either a pCR or rCR to systemic therapy, or the 27 who did not have a pCR to systemic therapy there is no significant difference between those in whom all metastatic deposits were treated and those who received PMRT without covering all sites of metastatic disease. Further, although the actuarial PFSx is higher in patients who achieve a complete radiographic response where all M1 disease was irradiated (80% vs 46%, P = NS), there are durable (>2-yr) progression free patients in both subsets where all sites were treated as well as those where not all sites were treated.

## Discussion

IBC remains an extremely aggressive form of breast cancer with a poor prognosis and high rates of distant disease recurrence. Given this, aggressive locoregional therapy more typical of definitive treatment is generally considered futile in metastatic patients. We report for the first time, durable stage IV NED status in highly selected patients with metastatic IBC treated with contemporary first-line chemotherapy regimens and aggressive locoregional therapy. We find that comprehensive treatment including neoadjuvant chemotherapy, modified radical mastectomy and comprehensive PMRT which includes all metastatic foci when feasible was associated with surprisingly durable NED status. In addition, pCR in the metastatic IBC setting is a powerful predictor for outcome. Our data suggest aggressive therapy even in patients with this advanced presentation is warranted in select cases.

The primary objective of our study was to retrospectively review our single-institutional experience in the treatment of stage IV IBC patients with metastatic disease that have completed neoadjuvant chemotherapy, modified radical mastectomy, and post-mastectomy radiation therapy to at least 50 Gy. This cohort included patients with visceral disease and multiple sites of disease and thus is not exclusively an oligometastatic cohort. We report a surprisingly high 5-yr OSx of 54% which is similar to outcomes reported for stage III IBC patients who complete similar therapy (Scotti et al. 2013). Surprisingly, sixteen patients remain stage IV NED, 12 with follow up over three years.

The second objective of our analysis was to identify prognostic factors to predict which patients may benefit from aggressive radiation therapy to limited sites of metastatic disease and to provide prognostic information to patients who have achieved stage IV NED status and completed multi-modality treatment. In our study, patients who were hormone receptor positive fared better in terms of both OS and PFS, which is highly consistent with previously published data supporting the use of appropriately targeted therapies (Harris et al. 2003; Hurley et al. 2006; Van Pelt et al. 2003). Patients who experienced a pCR also had more durable PFS after PMRT, regardless of the type of metastatic disease, suggesting that perhaps these patient’s tumors have more “favorable biology”. Interestingly, however, disease control outcomes were not different if all sites of distant disease could not be safely or feasibly irradiated. This conclusion also held true on subset analysis with patients who had a pCR only, a pathologic or radiographic CR, or no pCR.

**Figure 5:**
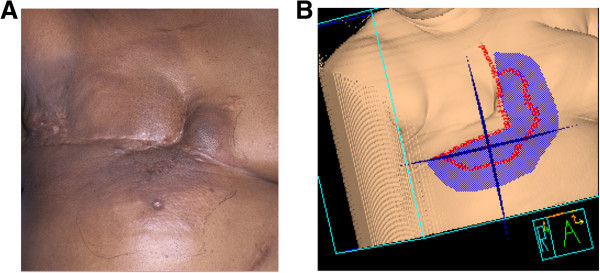
**Representative patient images. (A)** medical photograph of a patient with a local recurrence adjacent to the medial edge of the treatment field 6 months after completing neo-adjuvant systemic therapy, modified radical mastectomy, and post-mastectomy radiation therapy with outlined induration and erythema which were biopsy-proven as recurrence. **(B)** skin rendering of the patient’s second radiation treatment plan (after recurrence), with purple colorwash delineating the radiation treatment field.

We did have 5 patients diagnosed with a locoregional recurrence in our study cohort with one patient recurring prior to starting radiation therapy, and four recurring after PMRT for a 5-yr actuarial LRR of 14% at a median follow-up of 31 months. Nearly all of these patients recurred within a year of finishing radiation therapy which supports our clinical practice of following these patients closely immediately after they have finished treatment. This parallels the range of 8–22% described in the literature at 5 years in stage III IBC patients (Harris et al. 2003; Pisansky et al. 1992). Interestingly, three of the five failures encompassed the skin just outside of the medial border of the radiation field (Figure 5). This area is often treated conservatively in order to minimize radiation therapy to the contralateral breast; however, our review demonstrates the need for carefully weighing the radiation treatment margin with the probability of recurrence in this area as failure adjacent to the prior radiation field is significantly more difficult to treat, with increased morbidity. Radiotherapy has historically been associated with a reduction in LRR of 2/3 of the baseline risk although the influence of receptor type likely influences this (Kyndi et al. 2008). Even conservatively assuming a 50% reduction, these LRR rates suggest the baseline risk approaches 30% and highlights the value in preventing the morbidity of LRR in stage IV IBC. This is of particular interest in IBC given the potential for very morbid “en cuirasse”, armor-like local recurrence encircling the thorax.

As with any retrospective approach, there are limitations to this study. We had no a priori eligibility criteria for referral for surgery and PMRT. On opening a dedicated IBC clinic, patients were typically seen at presentation by all disciplines regardless of stage or prior to completing the staging which likely facilitated referral for local therapy when surgery was feasible. This of course indicates a selection bias which cannot be fully accounted for. Patients who progressed on chemotherapy or who were not amenable to margin negative mastectomy were excluded from this approach. Our study cohort is carefully selected to include only those patients with limited metastatic disease who also had a sufficient response to chemotherapy to warrant further local therapy. Our results in these stage IV patients corroborate multiple reports in stage III patients suggesting a pCR is prognostic and suggest response to chemotherapy may be as important as stage in this cohort. The role of a radiographic CR in our study remains unclear, and there are insufficient numbers to demonstrate value in radiating all involved sites. Furthermore, our study has a median follow up of 31 months after radiation therapy which somewhat limits our ability to compare our results with those of Stage III IBC patients who often have longer follow up.

Overall, the results are compelling and suggest that perhaps Stage IV IBC is only slightly further along the clinical spectrum than Stage III IBC which may harbor subclinical sites of disease that shortly thereafter manifest as disease progression (Tabbane et al. 1977). These results also suggest that T4d staging alone should not preclude patients with limited foci of metastatic disease from consideration of locoregional treatment, particularly those patients who have had a radiographic CR who therefore may be recognized as having a pCR at surgery, and those who are hormone receptor positive.

Further, local control is a significant issue for this population. Understanding the limitations of the data, we recommend treating all distant sites at the time of PMRT in stage IV IBC only when feasible and reasonably low risk and we recognize constraining the medial border of the chest wall field to midline may be inadequate margin on the medial scar in IBC. Further validation of these studies may suggest that increased incorporation of aggressive locoregional therapies in specific subsets of patients with metastatic IBC is warranted. Clearly some of these stage IV IBC patients have achieved a durable NED status potentially suggesting that some stage IV disease in IBC may represent extended regional lymphatic spread which may be potentially curable in the setting of effective chemotherapy. With further study this could also lead to the consideration of patients with focal metastatic disease or extended regional disease as a distinct staging category.

## Conclusions

The degree to which all gross disease needs to be addressed with locoregional therapy remains unclear. However, in the absence of randomized trials, these data suggest that aggressive, local therapy should be considered in select cases where the potential for locoregional control outweighs the risks of treatment. In addition, medial PMRT margins need to be generous, and targeting M1 disease should balance the potential for sterilization of all disease with toxicity and feasibility in all patients. We currently reserve this approach for patients who have no gross radiographic disease at the time of PMRT, or those in whom gross disease is easily and safely encompassed in limited fields to high or definitive dose.

## Abbreviations

NED: No evidence of disease
PMRT: Post-mastectomy radiation therapy
IBC: Inflammatory breast cancer
pCR: Pathologic complete response
rCR: Radiographic complete response
PFSx: Progression-free survival after post-mastectomy radiation therapy
LRR: Locoregional recurrence
LRC: Locoregional control
OS: Overall survival
OSx: Overall survival after post-mastectomy radiation therapy
CR: Complete response
ER: Estrogen receptor
IR: Interquartile range
Mo: Months
Yr: Years.
